# Overexpression of *Betaig-h3* gene downregulates integrin α5β1 and suppresses tumorigenicity in radiation-induced tumorigenic human bronchial epithelial cells

**DOI:** 10.1038/sj.bjc.6600304

**Published:** 2002-06-17

**Authors:** Y L Zhao, C Q Piao, T K Hei

**Affiliations:** Center for Radiological Research, College of Physicians and Surgeons of Columbia University, VC 11-218, 630 West 168th Street, New York, NY 10032, USA

**Keywords:** *Betaig-h3*, tumour suppression, human bronchial epithelial cells, integrin receptor

## Abstract

Interaction between cell and extracellular matrix plays a crucial role in tumour invasion and metastasis. Using an immortalised human bronchial epithelial (BEP2D) cell model, the study here shows that expression of *Betaig-h3* gene, which encodes a secreted adhesion molecule induced by transforming growth factor-β, is markedly decreased in several independently generated, radiation-induced tumour cell lines (TL1–TL5) relative to parental BEP2D cells. Transfection of *Betaig-h3* gene into tumour cells resulted in a significant reduction in tumour growth. While integrin receptor α5β1 was overexpressed in tumour cells, its expression was corrected to the level found in control BEP2D cells after *Betaig-h3* transfection. These data suggest that *Betaig-h3* gene is involved in tumour progression by regulating integrin receptor α5β1. The findings provide strong evidence that the *Betaig-h3* gene has tumour suppressor function in human BEP2D cell model and suggest a potential target for interventional therapy.

*British Journal of Cancer* (2002) **86**, 1923–1928. doi:10.1038/sj.bjc.6600304 www.bjcancer.com

© 2002 Cancer Research UK

## 

Tumour growth and metastasis is a multistep process involving cell adhesion, extracellular matrix (ECM) degradation and cell migration (Tlsty, 1998). The integrin superfamily consists of a major class of transmembrane glycoproteins that mediate cell–ECM and cell–cell adhesion (Giancotti and Ruoslahti, 1999). Loss or gain of expression of specific integrin has been implicated in malignant transformation, tumour progression and metastasis (Mizejewski, 1999). There is evidence that osteosarcoma cells that overexpress integrin α5β1 show reduced invasive potential (Giancotti and Ruoslahti, 1990). In contrast, upregulation of α5β1 has been shown to correlate with invasive phenotype in colon cancer and transitional cell carcinoma (Saito *et al*, 1996; Gong *et al*, 1997). Similarly, recent data show that high levels of integrin α6 in breast cancer and αvβ3 in melanoma correlate with tumour progression (Mukhopadhyay *et al*, 1999; Hofmann *et al*, 2000). Therefore, depending on the cell type and tumour model, expression of various integrin subunits may contribute either positively or negatively to the transformed phenotype.

Betaig-h3 is a secreted protein induced by transforming growth factor-β (TGF-β) in human adenocarcinoma cells as well as other human cell types (Skonier *et al*, 1992). Although transfection of *Betaig-h3* gene into CHO (Chinese Hamster Ovary) fibroblasts markedly reduces their ability to form tumours in nude mice (Skonier *et al*, 1994), its expression as well as regulation in human tumour has not been examined until now. There is evidence that mutations or altered expression of this gene are involved in corneal dystrophy and osteogenesis in human (Bron, 2000; Kim *et al*, 2000a). In addition, Betaig-h3 protein is a component of ECM in lung, bladder and skin (LeBaron *et al*, 1995; Billings *et al*, 2000a,b), which promotes adhesion and the spreading of dermal fibroblasts *in vitro* and mediates cell adhesion by interacting with α3β1 integrin in human corneal epithelial cells (Billings *et al*, 2000b; Kim *et al*, 2000b). These data suggest that Betaig-h3 protein is involved in cellular adhesion and imply an important role of this gene in the process of human tumour progression.

Although *in vitro* transformation studies with human cells are highly desirable in studying the molecular events associated with malignant conversion, such studies, thus far, have not been successful with primary human epithelial cells (Hei *et al*, 1994). Using papillomavirus-immortalised human bronchial epithelial (BEP2D) cells, we have previously shown that α-particles can malignantly transform these cells in a stepwise fashion before they become tumorigenic and metastatic in nude mice. It should be stated that although these cells are immortalised, they do not possess any other transformed phenotypes and only after carcinogen treatment, and extended subculturing, do transformed/tumorigenic phenotypes emerge in a sequential fashion (Hei *et al*, 1994, 1996). The BEP2D cell model is, therefore, useful in studying the genetic events involved in tumour progression. In the present study, we show that ectopic expression of *Betaig-h3* gene in radiation-induced tumour cells significantly suppresses their *in vivo* tumorigenicity. This finding provides strong evidence that *Betaig-h3* has tumour suppressor function in human BEP2D cells.

## MATERIALS AND METHODS

### Cell culture

Tumorigenic BEP2D cells were derived previously from treatment of exponentially growing BEP2D cells with a single 60 cGy dose of alpha-particles (Hei *et al*, 1996). Tumours larger than 1 cm in diameter were resected from nude mice and used to establish independently-generated cell lines (TL1–TL5). The BEP2D cells and tumour cell lines were maintained in serum-free LHC-8 medium supplemented with growth factors as described previously (Hei *et al*, 1994). Primary human bronchial epithelial (NHBE) cells were purchased from Clonetics (catalogue no: CC-2541) and grown in BEGM medium (Clonetics).

### cDNA array and Northern blotting

cDNA array (Clontech) was hybridised with ^32^P-labelled cDNA probes prepared by reverse transcription using 1 μg mRNA from control BEP2D and TL1 tumour cells as described previously (Zhao *et al*, 2001). The hybridisation signals were analysed by autoradiography and further quantified by phosphorimaging (ImageQuant software). The expression levels of β-actin and G3PDH housekeeping genes were used as standards for normalising the expression levels of other genes.

For Northern blot, 2.5 μg of mRNA was denatured and separated on a 1% denaturing agarose formaldehyde gel. The mRNAs were then transferred on nylon membrane (Millipore Corp., Bedford) by downward capillary blotting in 20×SSC (3 M NaCl, 0.3 M Na_3_Citrate·2H_2_O, pH 7.0) followed by UV cross-linking. Specific probe was generated by labelling of PCR-amplified cDNA fragments with [α-^32^P]dCTP using random primed DNA labelling kit (Boehringer, Mannheim). The membranes were pre-hybridised for 30 min and then hybridised with cDNA probe in ExpressHyb TM hybridisation solution (Clontech) for 8–12 h at 68°C. The blots were washed twice in 2×SSC, 0.1% SDS at room temperature for 15 min followed by washing twice in 0.2×SSC, 0.1% SDS at 55°C for 15 min. The membranes were exposed to Kodak BioMax film at –70°C for 12–72 h. The band intensities were evaluated by phosphorimaging and normalised to β-actin expression level.

All probes for Northern blot were acquired by PCR amplified gene fragments using the following primer sets: α5: 5′-AGAGCCAAAGTCTGCAGTTG-3′, 5′-CTGGAGGCTTGAGCTGAGCT-3′; β1: 5′-GTGTTCAGTGCAGAGCCTTCA-3′, 5′-CTTCGGATTGA CCACAGTTG-3′; β-actin: 5′-GTTGCTATCCAGGCTGTGC-3′, 5′-GCATCCTGTCGGCAATGC-3′.

### Cloning and sequencing of *Betaig-h3* cDNA

The first strand cDNA was synthesised from 0.2 μg poly(A)^+^ RNA isolated from NHBE cells using Superscript II reverse transcriptase and oligo(dT) primer (Gibco). Human *Betaig-h3* cDNA was then PCR-amplified using high-fidelity MasterAmp™ DNA polymerase (Epicenter, Madson, WI, USA) and synthetic primers (5′-GTTAAGCTTGCTTGCCCGTCGGTCGCTAGCT-3′, 5′-GCTCTAGAGCCTCCAAGCCACGTGTAGATGT-3′) that included *Hind*III and *Xba*I restriction enzyme recognition sites. The amplified whole length cDNA was subcloned into the *Hind*III and *Xba*I-digested pRc/CMV2 expression vector (Invitrogen). The sequence analysis showed that the protein sequence is 100% identical with that report in GeneBank (accession no: M77349) with the exception of several modified nucleotide sites such as 698 (C→G), 1667 (T→C) and 1118 (C→T).

### Transfection of TL1 tumour cells with *Betaig-h3* cDNA

TL1 tumour cells were plated at 1.5×10^6^ per 60 mm dish in serum-free LHC-8 medium. When 70–80% confluent, they were transfected with either pRc/CMV2-*Betaig-h3* or pRc/CMV2 (2 μg/dish) for 24 h using lipofectamin (Gibco) according to the manufacturer's instruction. The cells were split at 1 : 10 and cultured in the medium containing 500 μg ml^−1^ of the G418 (Gibco) for 21 days. Colonies were isolated using cloning ring and maintained in the presence of 300 μg ml^−1^ of G418.

### Immunoprecipitation and Western blotting

For screening the α5 and β1 integrin subunit expression, immunoprecipitations were carried out on surface-biotinylated cells as previously described (Trusolino *et al*, 1998). For analysis of *Betaig-h3* protein expression, conditioned medium was collected from confluent culture and the protein was then concentrated using SP sepharose (Amersham) and eluted using SDS sample buffer by boiling 5 min (Billings *et al*, 2000b). Protein concentrations were measured by Bio-Rad DC protein assay kit. Samples containing equal amounts of proteins were then fractionated by SDS–PAGE gel, transferred onto Hybond membrane, and immunoblotted with 1 : 1000 dilution of anti-Betaig-h3 human polyclonal antibody (kindly provided by Dr Paul C Billings). Peroxidase-conjugated anti-rabbit IgG was used to detect Betaig-h3 level by ECL procedures.

### *In vitro* growth rate

Growth curves of TL1 and *Betaig-h3*-transfected tumour cells were performed by plating 5×10^4^ cells into 25 cm^2^ flask. Cell numbers were determined using Coulter Counter. The results at each time point were the mean value of eight cultures from two independent experiments.

### Anchorage-independence growth and tumorigenicity in nude mice

Anchorage-independence assays were performed by plating the *Betaig-h3*-transfected and control BEP2D cells in 0.35% agar on the layer of 0.7% agar. Colonies ⩾10 cells in number were counted after 4 weeks. Tumorigenicity assay was performed as described previously (Hei *et al*, 1994). Briefly, *Betaig-h3*- or empty vector-transfected tumour cells were injected subcutaneously into nude mice at the left flanks. Tumours were palpated and measured with calipers and tumour volume calculated as (longest diameter× (shortest diameter)^2^)×0.5. Control animals were inoculated with either control BEP2D cells or with radiation-induced TL1 tumour cells. For each cell line, two independent experiments were performed.

## RESULTS

### *Betaig-h3* is downregulated in radiation-induced tumour cell lines

**Figure 1:**
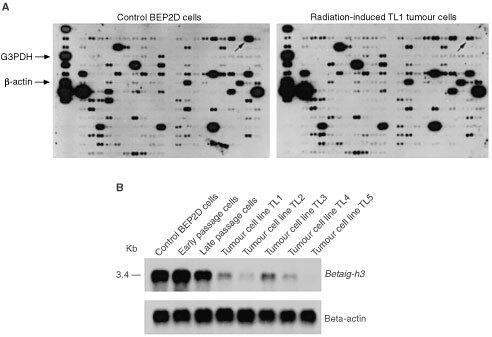
Differential expression of *Betaig-h3* gene in control BEP2D and radiation-induced tumour cells. (**A**) Human cytokine/receptor array results (Clontech Catalogue no: 7744–1). Arrows indicate the cDNA spots of *Betaig-h3* on the membrane. (**B**) Northern blot analysis of *Betaig-h3* gene in control BEP2D cells, early passage cells (1 week post radiation), late passage cells (just before inoculation into nude mice) and five tumour cell lines (TL1–TL5). The blots were hybridised to ^32^P-labelled *Betaig-h3* cDNA probes. After stripping, the membranes were rehybridised to human β-actin probe.

Tumorigenic BEP2D cells were established by exposing the non-tumorigenic, immortalised parental cells to a single 60 cGy dose of α-particles as described (Hei *et al*, 1996). A series of primary and secondary tumour cell lines (TL1–TL5) were established from tumour nodules developed in nude mice. By using cDNA array techniques, a series of genes were identified that were differentially expressed in radiation-induced tumour cells relative to parental BEP2D cells (Zhao *et al*, 2001). Among these genes, *Betaig-h3* expression was markedly decreased in tumour cells (Figure 1A

). The result was further confirmed by Northern blot using mRNAs obtained from different passages of transformed cells and five tumour cell lines (Figure 1B). In early-passaged cells (1 week after radiation), no change in *Betaig-h3* expression was found when compared with control BEP2D cells. However, the expression of *Betaig-h3* was downregulated by 2.4-fold in late-passaged cells (just before inoculating into nude mice) and between 7.5–9-fold in all five tumour cell lines examined. These results indicate that decreased expression of *Betaig-h3* gene might be related to the acquisition of malignant phenotype in BEP2D cells.

### Overexpression of *Betaig-h3* gene in tumour cells suppresses their colony-forming efficiency in soft agar and tumorigenicity in nude mice

**Figure 2:**
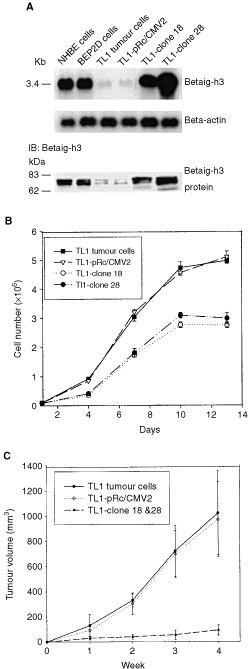
(**A**) mRNA and protein levels of *Betaig-h3* gene determined by Northern blot and immunoblotting (IB) in normal NHBE, control BEP2D, TL1 and *Betaig-h3*-transfected tumour cells. (**B**) *In vitro* growth rate of parental TL1 and *Betaig-h3*-transfected tumour cells. Data represent mean±s.d. of eight culture flasks from two independent experiments. (**C**) Inhibition of tumour growth by *Betaig-h3* transfection relative to vector alone and parental TL1 tumour cells. Results are expressed as the mean±s.d. of 8–9 independent tumours.

To examine the significance of *Betaig-h3* downregulation in malignant conversion, we recovered the expression of *Betaig-h3* gene in a representative tumour cell line (TL1) with pRc/CMV2-Betaigh3 vector. Two G418-resistant colonies (TL1-clones 18 and TL1-clone 28) that expressed different levels of *Betaig-h3* were chosen for further studies. From the Northern and Western blot results (Figure 2A

), the parental TL1 and TL1-pRc/CMV2 cells (vector control) expressed similar levels of *Betaig-h3*, which were lower than control BEP2D cells. After *Betaig-h3* transfection, the expression of this gene in TL1-clone 18 was recovered to a level similar to that of control BEP2D cells, whereas TL1-clone 28 had a four-fold higher expression level. Expression of the *Betaig-h3* gene in primary human bronchial epithelial (NHBE) cells and control BEP2D cells, on the other hand, was similar both at the mRNA and protein levels (Figure 2A).

TL1-clone 18 and TL1-clone 28 cells grew much slower and showed lower saturation density than parental TL1 tumour cells (Figure 2B). The doubling time of cells transfected with the empty vector was 32 h, which was similar to that of TL1 tumour cells. In contrast, clone 18 and 28 cell lines grew slower than TL1, with doubling times of about 55 h. We also checked their colony-forming efficiency in soft agar (Table 1

**Table 1:**
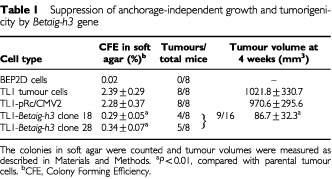
Suppression of anchorage-independent growth and tumorigenicity by *Betaig-h3* gene

). The result showed that there was no significant difference between parental TL1 and TL1-pRc/CMV2 cells (2.39 and 2.28%, respectively with *P*>0.05). However, TL1-clone 18 and TL1-clone 28 cells resulted in a significantly lower ability of anchorage independent growth with colony-forming efficiency in agar of 0.29 and 0.34%, respectively (*P*<0.01).

To determine whether ectopic expression of the *Betaig-h3* gene suppresses tumour formation *in vivo*, 5×10^6^ of each of the following cell types were subcutaneously injected into nude mice: control BEP2D cells, TL1 tumour cells, TL1-pRc/CMV2 and *Betaig-h3*-transfected cells (clone 18 and 28). The tumour volumes were measured weekly during the experiments. As shown in Table 1, no tumours (zero out of eight mice) were found in parental BEP2D cells after monitoring for more than 20 weeks. However, eight out of eight mice that were injected with either TL1 or TL1-pRc/CMV2 tumour cells developed progressively growing tumours with average volumes of 1021.8±330.7 mm^3^ and 970.6±295.6 mm^3^, respectively. In contrast, four out of eight mice with TL1-clone 18 and five out of eight mice with TL1-clone 28 cells formed only small nodules. The volume, which averaged 86.7±32.3 mm^3^, was significantly smaller than that of parental TL1 tumour cells (*P*<0.01). Meanwhile, tumour growth was significantly suppressed in tumour cells after *Betaig-h3* transfection (Figure 2C).

### *Betaig-h3* gene is related to the expression level of integrin receptor α5β1

**Figure 3:**
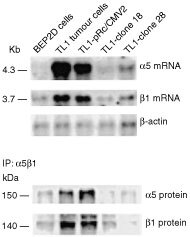
mRNA and protein levels of α5β1 determined by Northern blot and immunoprecipitation (IP) in TL1 and *Betaig-h3*-transfected tumour cells.

Previous studies have suggested that Betaig-h3 protein affect cell–ECM interaction through regulation of integrin receptor (LeBaron *et al*, 1995; Billings *et al*, 2000a,b). Using cDNA array, it was found that α5β1 integrin receptor was overexpressed in radiation-induced tumour cells (data not shown). To determine whether the expression of integrin receptor α5β1 correlated with *Betaig-h3* gene expression, we checked its mRNA and protein levels in *Betaig-h3* transfected tumour cells. As shown in Figure 3

, expression of α5 and β1 subunits was five- and three-fold higher, respectively, in parental TL1 and TL1-pRc/CMV2 cells than in control BEP2D cells. However, after transfecting *Betaig-h3* gene into TL1 tumour cells, expression of α5β1 integrin (clone 18 and 28 cells) decreased to level of control BEP2D cells. This data were further confirmed by immunoprecipitation using monoclonal antibody for α5 and β1. We further checked the mRNA expression of integrin subunits α1-α4, α6, αv and β2-β3. No significant changes in their expression were found among control BEP2D, TL1 tumour cells and *Betaig-h3*-transfected TL1 tumour cells (data not shown).

## DISCUSSION

In an attempt to identify genes involved in the progression of lung carcinoma, cDNA arrays were used to screen differentially expressed genes between control BEP2D and radiation-induced tumour cells. Altered expression of a series of genes that controlled cellular growth and differentiation between these two cell models was found (Zhao *et al*, 2001), with *Betaig-h3* gene notably downregulated in tumour cells, a finding that was further confirmed in five tumour cell lines by Northern blot. Previous studies have shown that *Betaig-h3* gene is significantly reduced in embryonal rhabdomyosarcoma cell lines and mesenchymal tumours (Genini *et al*, 1996; Schenker and Trueb, 1998), suggesting that *Betaig-h3* may have an important role in human cancer. Although overexpression of this gene in CHO fibroblast cells leads to a marked decrease in their ability to form tumour in nude mice (Skonier *et al*, 1994), little is known about its regulation in tumour progression of human tissues. In this study, we provide evidence that ectopic expression of *Betaig-h3* in TL1 tumour cells significantly inhibits colony-forming efficiency in soft agar, and tumour growth in nude mice relative to parental tumour cells. This is the first evidence that *Betaig-h3* gene has tumour suppressor function in a human epithelium-derived tumour model.

*Betaig-h3* is a secreted protein that promotes the adhesion of dermal fibroblasts and corneal epithelial cells (LeBaron *et al*, 1995; Billings *et al*, 2000a,b;). These findings imply that *Betaig-h3* gene affects cell–ECM interaction by regulating integrin receptors. This study confirms that acquisition of tumorigenic phenotype of BEP2D cells is accompanied by an increased expression of α5β1 integrin receptor at both the mRNA and protein levels. Ectopic expression of *Betaig-h3* gene in tumorigenic cells (TL1) led to downregulation of integrin and suppression of tumorigenicity. The data suggest that *Betaig-h3* gene is involved in the tumorigenic process by regulating α5β1 expression. The observation is consistent with other reports that α5β1, while undetectable in normal lung epithelial, is significantly elevated in SV40 large T-transformed human bronchial epithelial cells (Albelda and Buck, 1990; Schiller and Bittner, 1995). In non-small lung carcinoma cells, higher levels of α5β1 expression represents a negative prognostic factor (Adachi *et al*, 2000). Similar results have also been shown with other human tissues that high levels of α5β1 integrin is associated with more malignant phenotype in melanoma, transitional and colon cell carcinomas (Saito *et al*, 1996; Gong *et al*, 1997; Beliveau *et al*, 2000). The α5β1 integrin favours cell survival and protects cells from apoptosis *in vitro* via upregulation of anti-apoptotic Bcl-2, whereas resistance to apoptosis is a feature of many malignant cells (Zhang *et al*, 1995). These data, together with our results, suggest a key role for α5β1 overexpression in tumorigenicity of human bronchial epithelial cells. Although there is evidence that the Betaig-h3 protein mediates cell adhesion by interacting with α3β1 (Kim *et al*, 2000b), integrins are expressed in a cell-type- and stage-specific manner (Ruoslahti, 1999). Examples of cell-type-specific integrins include αIIβ3 in platelets and α6β4 in epithelial cells. One group of integrins is associated with migration and proliferation in various types of cells. These ‘emergency integrins’ which include α5β1, αvβ3, and αvβ6 (Sheppard, 1996) are particularly important in cancer. However, no differential expression of α1-α4, α6, αv and β2-β3 integrin subunits was found between *Betaig-h3* transfected and parental TL1 tumour cells. The data suggest that *Betaig-h3* gene is involved in tumour progression of human bronchial epithelial cell model by regulating integrin receptor α5β1.

Altered cell–matrix interaction is an essential prerequisite step in the invasive and metastatic cascade (Hart and Saini, 1992). Our finding that normal NHBE and immortalised BEP2D cells exhibit similar levels of *Betaig-h3* expression suggests that loss of its expression occur during late stage of tumour progression. Previous data show that chromosome 5q31, where *Betaig-h3* gene has been regionally mapped to, is often deleted in leukaemias, myelodysplastic syndromes and many human cancer such as renal cell, oesophageal and lung carcinomas (Peralta *et al*, 1998; Wu *et al*, 1998; Brezinova *et al*, 2000). These findings suggest that deletion of *Betaig-h3* gene is a frequent event in human cancer. The question of whether reexpression of *Betaig-h3* gene in human tumour cell lines may result in suppression of tumorigenicity is currently under investigation. Our present finding suggest that *Betaig-h3* gene could be a novel diagnostic marker of tumour metastasis and a potential target for cancer therapy.

## References

[bib1] AdachiMTakiTHigashiyamaMKohnoNInufusaHMiyakeM2000 Significance of integrin alpha5 gene expression as a prognostic factor in node-negative non-small cell lung cancer Clin Cancer Res 6196101 10656437

[bib2] AlbeldaSMBuckCA1990 Integrins and other cell adhesion molecules FASEB J 41128682880 2199285

[bib3] BeliveauABerubeMRousseauAPelletierGGuerinSL2000 Expression of integrin alpha5beta1 and MMPs associated with epithelioid morphology and malignancy of uveal melanoma Invest Ophthalmol Vis Sci 41823632372 10892885

[bib4] BillingsPCHerrickDJHowardPSKucichUEngelsbergBNRosenbloomJ2000a Expression of *Betaig-h3* by human bronchial smooth muscle cells: localization to the extracellular matrix and nucleus Am J Respir Cell Mol Biol 223352359 10696072 10.1165/ajrcmb.22.3.3732

[bib5] BillingsPCHerrickDJKucichUEngelsbergBNAbramsWRMacarakEJRosenbloomJHowardPS2000b Extracellular matrix and nuclear localization of betaig-h3 in human bladder smooth muscle and fibroblast cells J Cell Biochem 792261273 10967553 10.1002/1097-4644(20001101)79:2<261::aid-jcb90>3.0.co;2-#

[bib6] BrezinovaJZemanovaZCermakJMichalovaK2000 Fluorescence in situ hybridization confirmation of 5q deletions in patients with hematological malignancies Cancer Genet Cytogenet 11714549 10700866 10.1016/s0165-4608(99)00142-9

[bib7] BronAJ2000 Genetics of the corneal dystrophies: what we have learned in the past twenty-five years Cornea 195699711 11009322 10.1097/00003226-200009000-00015

[bib8] GeniniMSchwalbePSchollFASchaferBW1996 Isolation of genes differentially expressed in human primary myoblasts and embryonal rhabdomyosarcoma Int J Cancer 664571577 8635876 10.1002/(SICI)1097-0215(19960516)66:4<571::AID-IJC24>3.0.CO;2-9

[bib9] GiancottiFGRuoslahtiE1999 Integrin signaling Science 285 10281032 10446041 10.1126/science.285.5430.1028

[bib10] GiancottiFGRuoslahtiE1990 Elevated levels of the alpha 5 beta 1 fibronectin receptor suppress the transformed phenotype of Chinese hamster ovary cells Cell 605849859 2155708 10.1016/0092-8674(90)90098-y

[bib11] GongJWangDSunLZborowskaEWillsonJKBrattainMG1997 Role of alpha 5 beta 1 integrin in determining malignant properties of colon carcinoma cells Cell Growth Differ 818390 8993837

[bib12] HartIRSainiA1992 Biology of tumor metastasis Lancet 339 14531457 1376386 10.1016/0140-6736(92)92039-i

[bib13] HeiTKPiaoCQWilleyJCThomasSHallEJ1994 Malignant transformation of human bronchial epithelial cells by radon-simulated alpha-particles Carciongenesis 153431437 10.1093/carcin/15.3.431 8118924

[bib14] HeiTKPiaoCQHanESutterTWillyJC1996 Radon-induced neoplastic transformation of human bronchial epithelial cells Radiat Oncol Invest 3 398403

[bib15] HofmannUBWestphalJRWaasETBeckerJCRuiterDJvan MuijenGN2000 Coexpression of integrin alphavbeta3 and matrix metallo-proteinase-2 (MMP-2) coincides with MMP-2 activation: correlation with melanoma progression J Invest Dermatol 1154625632 10998134 10.1046/j.1523-1747.2000.00114.x

[bib16] KimJEKimEHHanEHParkRWParkIHJunSHKimJCYoungMFKimIS2000a TGF-beta-inducible cell adhesion molecule, betaig-h3, is downregulated in melorheostosis and involved in osteogenesis J Cell Biochem 772169178 10723084 10.1002/(sici)1097-4644(20000501)77:2<169::aid-jcb1>3.0.co;2-l

[bib17] KimJEKimSJLeeBHParkRWKimKSKimIS2000b Identification of motifs for cell adhesion within the repeated domains of transforming growth factor-beta-induced gene, betaig-h3 J Biol Chem 275403090730915 10906123 10.1074/jbc.M002752200

[bib18] LeBaronRGBezverkovKIZimberMPPavelecRSkonierJPurchioAF1995 Beta IG-H3, a novel secretory protein inducible by transforming growth factor-beta, is present in normal skin and promotes the adhesion and spreading of dermal fibroblasts in vitro J Invest Dermatol 1045844849 7738366 10.1111/1523-1747.ep12607024

[bib19] MizejewskiGJ1999 Role of integrins in cancer: survey of expression patterns Proc Soc Exp Biol Med 2222124138 10564536 10.1177/153537029922200203

[bib20] MukhopadhyayRTheriaultRLPriceJE1999 Increased levels of alpha6 integrins are associated with the metastatic phenotype of human breast cancer cells Clin Exp Metastasis 174325332 10545019 10.1023/a:1006659230585

[bib21] PeraltaRCCassonAGWangRNKeshavjeeSRedstonMBapatB1998 Distinct regions of frequent loss of heterozygosity of chromosome 5p and 5q in human esophageal cancer Int J Cancer 785600605 9808529 10.1002/(sici)1097-0215(19981123)78:5<600::aid-ijc12>3.0.co;2-1

[bib22] RuoslahtiE1999 Fibronectin and its integrin receptors in cancer Advances in Cancer Research 76 120 10218097 10.1016/s0065-230x(08)60772-1

[bib23] SaitoTKimuraMKawasakiTSatoSTomitaY1996 Correlation between integrin alpha 5 expression and the malignant phenotype of transitional cell carcinoma Br J Cancer 733327331 8562338 10.1038/bjc.1996.57 PMC2074419

[bib24] SchenkerTTruebB1998 Down-regulated proteins of mesenchymal tumor cells Exp Cell Res 2391161168 9511734 10.1006/excr.1997.3896

[bib25] SchillerJHBittnerG1995 Loss of the tumorigenic phenotype with in vitro, but not in vivo, passaging of a novel series of human bronchial epithelial cell lines: possible role of an alpha 5/beta 1-integrin-fibronectin interaction Cancer Res 552462156221 8521416

[bib26] SkonierJNeubauerMMadisenLBennettKPlowmanGDPurchioAF1992 cDNA cloning and sequence analysis of beta Betaig-h3, a novel gene induced in a human adenocarcinoma cell line after treatment with transforming growth factor-beta DNA Cell Biol 117511522 1388724 10.1089/dna.1992.11.511

[bib27] SkonierJBennettKRothwellVKosowskiSPlowmanGWallacePEdelhoffSDistecheCNeubauerMMarquardtH1994 Betaig-h3: a transforming growth factor-beta-responsive gene encoding a secreted protein that inhibits cell attachment in vitro and suppresses the growth of CHO cells in nude mice DNA Cell Biol 136571584 8024701 10.1089/dna.1994.13.571

[bib28] SheppardD1996 Epithelial integrins Bioessays 188655660 8760339 10.1002/bies.950180809

[bib29] TlstyTD1998 Cell-adhesion-dependent influences on genomic instability and carcinogenesis Curr Opin Cell Biol 105647653 9818176 10.1016/s0955-0674(98)80041-0

[bib30] TrusolinoLSeriniGCecchiniGBesatiCAmbesi-ImpiombatoFSMarchisioPCDe FilippiR1998 Growth factor-dependent activation of alphavbeta3 integrin in normal epithelial cells: implications for tumor invasion J Cell Biol 142411451156 9722624 10.1083/jcb.142.4.1145 PMC2132885

[bib31] WuXZhaoYKempBLAmosCISicilianoMJSpitzMR1998 Chromosome 5 aberrations and genetic predisposition to lung cancer Int J Cancer 795490493 9761118 10.1002/(sici)1097-0215(19981023)79:5<490::aid-ijc8>3.0.co;2-w

[bib32] ZhaoYLPiaoCQHallEJHeiTK2001 Mechanisms of radiation-induced neoplastic transformation of human bronchial epithelial cells Radiat Res 1551 Pt2230234 11121239 10.1667/0033-7587(2001)155[0230:morint]2.0.co;2

[bib33] ZhangZVuoriKReedJCRuoslahtiE1995 The alpha 5 beta 1 integrin supports survival of cells on fibronectin and up-regulates Bcl-2 expression Proc Natl Acad Sci USA 921361616165 7541142 10.1073/pnas.92.13.6161 PMC41662

